# Epicardial Adipose Tissue and Atrial Fibrillation Recurrence following Catheter Ablation: A Systematic Review and Meta-Analysis

**DOI:** 10.3390/jcm12196369

**Published:** 2023-10-05

**Authors:** Ioannis Anagnostopoulos, Maria Kousta, Charalampos Kossyvakis, Nikolaos Taxiarchis Paraskevaidis, Dimitrios Vrachatis, Spyridon Deftereos, Georgios Giannopoulos

**Affiliations:** 1 Cardiology Department, Athens General Hospital “G. Gennimatas”, 11527 Athens, Greececkossyvakis@gmail.com (C.K.);; 2 2nd Department of Cardiology, National and Kapodistrian University of Athens, 15772 Athens, Greece; 3 3rd Department of Cardiology, Aristotle University of Thessaloniki, 54124 Thessaloniki, Greece

**Keywords:** atrial fibrillation, catheter ablation, recurrence, epicardial adipose tissue, epicardial fat

## Abstract

(1)Introduction: Catheter ablation has become a cornerstone for the management of patients with atrial fibrillation (AF). Nevertheless, recurrence rates remain high. Epicardial adipose tissue (EAT) has been associated with AF pathogenesis and maintenance. However, the literature has provided equivocal results regarding the relationship between EAT and post-ablation recurrence.(2) Purpose: to investigate the relationship between total and peri-left atrium (peri-LA) EAT with post-ablation AF recurrence. (3) Methods: major electronic databases were searched for articles assessing the relationship between EAT, quantified using computed tomography, and the recurrence of AF following catheter ablation procedures. (4) Results: Twelve studies (2179 patients) assessed total EAT and another twelve (2879 patients) peri-LA EAT. Almost 60% of the included patients had paroxysmal AF and recurrence was documented in 34%. Those who maintained sinus rhythm had a significantly lower volume of peri-LA EAT (SMD: −0.37, 95%; CI: −0.58–0.16, I2: 68%). On the contrary, no significant difference was documented for total EAT (SMD: −0.32, 95%; CI: −0.65–0.01; I2: 92%). No differences were revealed between radiofrequency and cryoenergy pulmonary venous isolation. No publication bias was identified. (5) Conclusions: Only peri-LA EAT seems to be predictive of post-ablation AF recurrence. These findings may reflect different pathophysiological roles of EAT depending on its location. Whether peri-LA EAT can be used as a predictor and target to prevent recurrence is a matter of further research.

## 1. Introduction

Atrial fibrillation (AF) is the most common tachyarrhythmia in the clinical practice [1]. It affects approximately 33.5 million people worldwide and its prevalence in Europe is expected to double in patients older than 55 years old by 2060 [2,3]. AF results in complications, such as stroke and thromboembolism, while it is associated with high morbidity and mortality rates [4,5].

Pulmonary venous isolation is the most acceptable targeted method for the catheter ablation of drug-refractory AF [6]. Nevertheless, the 12-month success rates of this method remain about 65% [7]. In an effort to increase the proportion of patients who will maintain normal sinus rhythm, there is an ongoing effort to identify, and whenever possible treat, factors that are associated with arrhythmia recurrence. 

Epicardial adipose tissue (EAT) is an active organ, in direct contact with the myocardium, which contains ganglionated plexuses and adipocytes [8]. This tissue has a rich secretome, including proinflammatory and prothrombotic cytokines, such as tumor necrosis factor-a and interleukin-6 [9]. Several studies have established the role of EAT in metabolic syndrome, diabetes mellitus and cardiovascular disease [10,11,12]. Furthermore, a recent investigation supports an association between increased levels of EAT and prevalent AF [13,14]. The increasing body of evidence suggesting that this relationship is not just an epiphenomenon [15] led to the investigation of the role of EAT surrounding left atrium (peri-LA EAT), which was also found to be associated with incident and prevalent AF [16,17]. 

These findings, along with the widespread use of imaging technologies to better guide the management approach of AF patients [18], motivated several researchers to examine the role of EAT in arrhythmia recurrence following catheter ablation. Nevertheless, their findings have been equivocal [19,20]. Thus, in this systematic review and meta-analysis we sought to gather the existing literature findings and investigate the predictive role of both total and peri-LA EAT on AF recurrence after pulmonary venous isolation.

## 2. Methods

This systematic review and meta-analysis was performed in accordance with the PRISMA guidelines [21]. The predefined protocol was registered in PROSPERO database (CRD42022361738).

### 2.1. Search Strategy

A systematic search of the literature was performed, up to September 2022, in three databases: Medline (via PubMed) and Scopus, using the following search strategy based on keywords ((epicardial fat) OR (epicardial adipose) OR (epicardial adiposity) AND (atrial fibrillation)). An additional hand search was also performed using the references of the articles that were identified as relevant (snowball strategy). No date restriction was used. Articles not available in English were excluded.

### 2.2. Study Selection

All articles obtained through the initial search were screened by two independent reviewers (M.K. and N.P.) on title and abstract level. Potentially eligible studies were further reviewed for inclusion in the final analysis based on the full text. Any disagreements were resolved after consensus with an expert (G.G.). The eligibility criteria for inclusion were (a) prospective or retrospective studies including patients with paroxysmal or/and persistent AF who underwent endocardial pulmonary venous isolation with radiofrequency catheter ablation or cryoballoon ablation, (b) preprocedural quantification of EAT (either total or peri-LA) using computed tomography and (c) a follow-up of at least 6 months for the detection of post-ablation recurrence. Recurrence was defined as AF/AT episodes, allowing a blanking period of 3 months, in most of the studies.

### 2.3. Data Extraction

Data of interest were extracted in a predesigned Microsoft Office Excel 2007 form by two independent reviewers (M.K. and N.P.) and crosschecked for any disagreements, which were resolved after consensus with a senior (G.G.). In the case of missing data or any other uncertainties, we contacted authors electronically (via email) to obtain the required data. 

### 2.4. Risk of Bias

Risk of bias, concerning the outcomes of interest, was assessed in duplicate with the National Institutes of Health Quality Assessment Tool for Observational Cohort and Cross-Sectional Studies [22], modified to better fit our studies (Supplementary Table S1). Disagreements were resolved by consensus. Studies with an overall score of ≥80% were considered as high quality. Publication bias was evaluated visually using funnel plots and statistically with the Egger’s test [23].

### 2.5. Statistical Analysis

Continuous variables were summarized as the mean (standard deviation). When the available data were summarized as the median with interquartile range, the median was assumed as the mean and the standard deviation was obtained by dividing the interquartile range by 1.35. Topool the outcomes of interest, the standardized mean differences (SMD) with the corresponding 95% confidence interval (CI) in pre-ablation total and peri-LA EAT, between patients with and without post-ablation AF recurrence, we performed a meta-analysis based on aggregate data. To allow for expected effect size dispersion between studies (based on intra/inter-observer variation, different methodology/software used and different sample synthesis), a random effects (DerSimonian-Laird) model was adopted. Heterogeneity was assessed using I^2^, with values between 50% and 90% representing substantial heterogeneity [24]. To explore heterogeneity, sensitivity analysis (based on the study’s overall quality) and subgroup analysis were performed. Moreover, meta-regression analysis was performed to explore the confounding effect of age, body mass index and male gender, as well as the number of patients with paroxysmal AF and hypertension. All analyses were performed using R Foundation software, version 4.1.2.

## 3. Results

### 3.1. Search Results and Studies Characteristics

**Figure 1:**
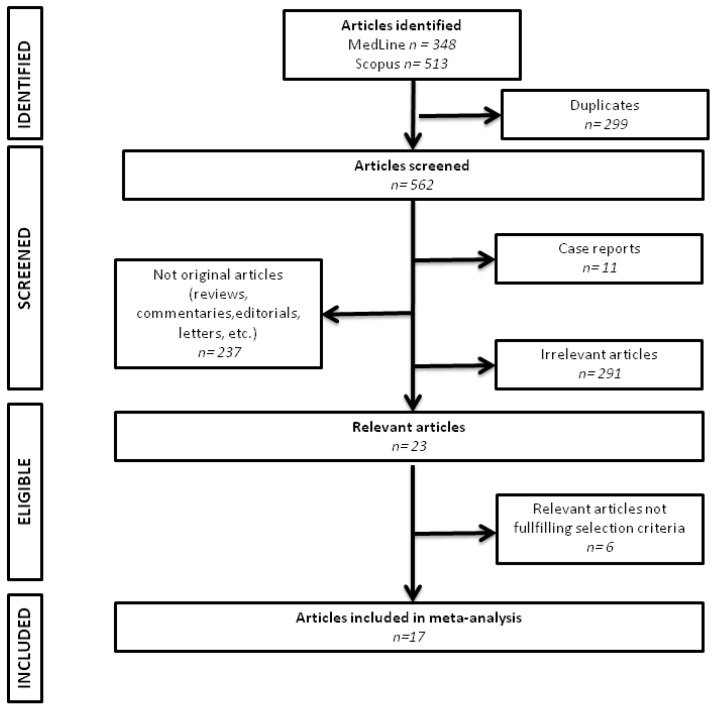
Flowchart of studies selection.

861 articles were retrieved by the electronic search. The flow of study selection is depicted in Figure 1. Fifteen articles were considered to be eligible and were included in the final analysis [19,20,25,26,27,28,29,30,31,32,33,34,35,36,37]. We analyzed 3035 patients (67% males). The mean (SD) age and body mass index were 59.8 (10.6) years and 25.71 (3.79), respectively. Among them, almost 60% had PAF, while the main comorbidity was hypertension. For the quantification of EAT, the vast majority of the included studies used a semi-automated protocol, recognizing adipose tissue by assigning threshold attenuation values between −200 and −50 Hounsfield units. The pooled mean pre-ablation total EAT volume was 121.81 ± 53.13 mL (assessed in twelvestudies), while the mean peri-LA EAT was 24.6 ± 15.61 mL (assessed in twelvestudies). In the majority of studies, pulmonary venous isolation was performed using radiofrequency ablation (ninestudies; used additional ablation procedures); cryoablation only was used in threestudies, while in the rest of them both techniques were performed based on the preference of the treating electrophysiologists. The mean follow-up ranged from 7.6 to 19.5 months. Except two studies, all the others were performed in Asia. AF recurrence occurred in 34% of the patients analyzed. The studies’ characteristics are summarized in Table 1.

### 3.2. Bias Assessment

Five studies were classified as being high quality, while the rest presented a moderate risk of bias (i.e., a score between 60 and 80%), mainly because of the lack of power analysis (Supplementary Table S2). A visual assessment of funnel plots did not reveal signs of publication bias (Supplementary Figures S1 and S2). In parallel, Egger’s test demonstrated no significant small study effect (*p* = 0.82 for total EAT and *p* = 0.19 for peri-LA EAT).

### 3.3. Total EAT and AF Recurrence

**Figure 2:**
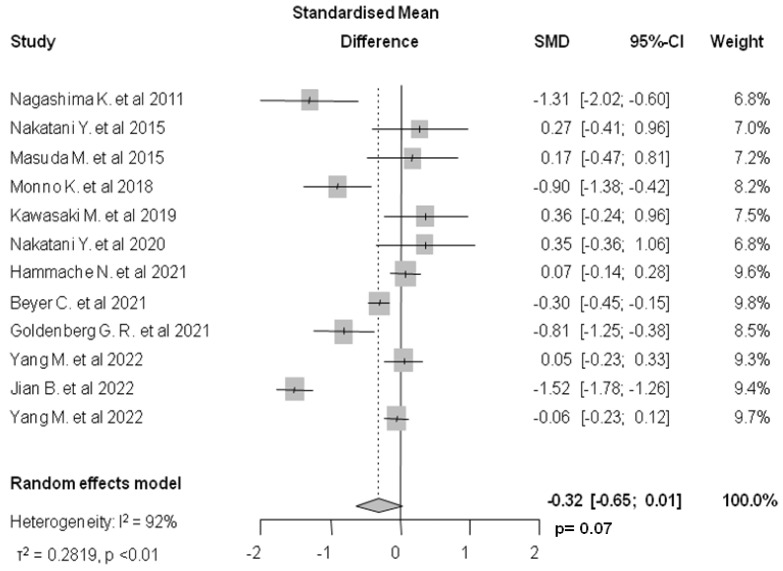
Forest plot of pre-ablation total EAT standardized mean difference between patients with and without AF recurrence. EAT: epicardial adipose tissue, AF: atrial fibrillation [19,20,25,28,29,31,32,33,34,35,36,37].

Twelve studies (2179 patients) analyzed the relationship between total EAT and AF recurrence. The mean (SD) pre-ablation total EAT was 124.28 (55.82) mL in the group of patients who experienced arrhythmia recurrence and 120.3 (49.86) mL in the non-recurrence group. Meta-analysis of aggregate data revealed a non-statistically significant standardized mean difference (SMD: −0.32, 95%, CI: −0.65–0.01, 95%, I^2^: 92%, *p* = 0.07, Figure 2).

No significant confounding effect of body mass index, gender or the number of patients with hypertension and paroxysmal AF was revealed in the meta-regression analysis. On the contrary, increasing age was found to significantly increase SMD (beta coefficient = 0.1, *p* = 0.01, residual I^2^: 89.9%).

A sensitivity analysis including only high quality studies revealed similar results (five studies, SMD: −0.03, 95%, CI: −0.38–0.32, I^2^: 75%). A subgroup analysis based on the method used for pulmonary venous isolation (Supplementary Figure S3), the age of the included patients (using 60 years as the cut-off) and the duration of the follow-up did not significantly change the results above.

### 3.4. Peri-LA EAT and AF Recurrence

**Figure 3:**
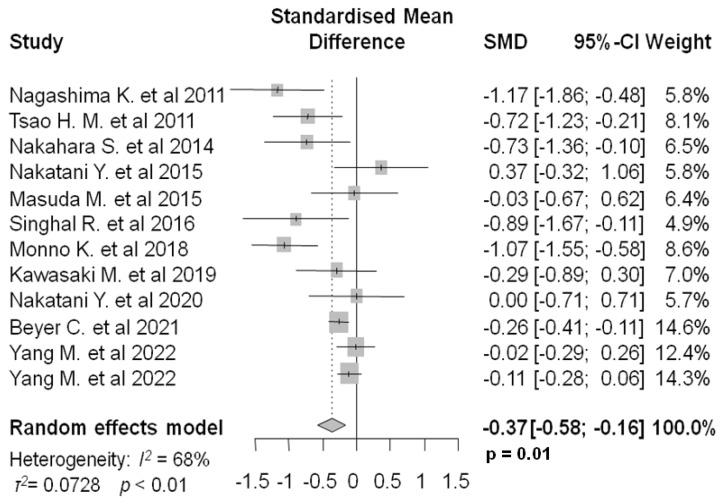
Forest plot of pre-ablation peri-LA EAT standardized mean difference between patients with and without AF recurrence. LA: left atrium, EAT: epicardial adipose tissue, AF: atrial fibrillation [19,20,25,26,27,28,29,30,31,32,35,37].

In the twelve studies (2879 patients) analyzing peri-LA EAT, the mean pre-ablation values in the groups of patients with and without AF recurrence were 26.11 (18.22) mL and 23.93 (14.35) mL, respectively. Meta-analysis revealed a statistically significant standardized mean difference (SMD: −0.37, 95%, CI: −0.58–−0.16, I2: 68%, *p* = 0.0006, Figure 3) between the two groups.

Because only three studies were of low risk of bias, no sensitivity analysis was performed based on the overall quality. A subgroup analysis based on the method used for pulmonary venous isolation (cryoenergy versus radiofrequency) and meta-regression analysis, using the same patients’ characteristics as above, did not reveal any significant sources of heterogeneity. In the subgroup analysis based on the duration of follow-up, a statistically significant difference was demonstrated only for the subgroup of studies which followed patients for more than 12 months (Supplementary Figure S4). 

Finally, because of the findings above, we performed an additional, not preplanned, analysis, where we included only studies that provided information for both total and peri-LA EAT. In this analysis of ninestudies with 2023 patients, again only peri-LA and not total EAT was associated with AF recurrence (Supplementary Figures S5 and S6).

## 4. Discussion

This study is a meta-analysis assessing the relationship between EAT and AF recurrence following catheter ablation. We analyzed both total and peri-LA EAT, quantified using computed tomography. The main finding is that only peri-LA, and not total, EAT is associated with arrhythmia recurrence. Patients with higher peri-LA EAT volume seem to have less chance atmaintaining sinus rhythm following pulmonary venous isolation. According to the subgroup analysis, this association seems to be mainly driven by studies that followed patients beyond 12 months, while no association with the body mass index was documented. Interestingly, in the analysis restricted to studies that assessed both total and peri-LA EAT, the results remained similar. Thus, the initial findings do not seem to be attributed to discrepancies between the studies’ population and methodology. All of the results should be interpreted with caution because of the existing heterogeneity. 

Previous meta-analyses in this topic have provided equivocal results, especially regarding the role of total EAT. Shamloo et al. synthesized four studies and concluded that both total and peri-LA EAT volumes differed significantly between patients with and without recurrence [38]. On the other hand, Chen et al., in a subgroup analysis of four studies assessing total EAT volume, did not prove a statistically significant relationship with AF recurrence [39]. Our analysis, which incorporated a significantly higher number of studies, comes to strengthen our knowledge regarding the role of EAT in post-ablation recurrence.

Various researchers have examined the role of EAT in the pathogenesis of AF. Nowadays, it is well established that EAT is an active organwith rich secretome leading to adverse mechanical and electrophysiological cardiac remodeling. The production of various profibrotic factors promotes tissue fibrosis [40,41]. This process is further enhanced by the secretion of inflammatory cytokineswhichlead to myocardial infiltration viamacrophages and lymphocytes [9,42,43]. Beyond this, inflammation itself seems to mediate a direct subepicardial fibrofatty infiltration [41,44]. These mechanisms may be responsible for the documented association between increased EAT and impaired systolic and diastolic atrial function [45,46], which, along with the increased levels of inflammation, are known to promote post-ablation AF recurrence [47,48].

Beyond the mechanical remodeling, EAT has been also associated with electrical remodeling. Adipocytes seem to increase sodium and potassium depolarizing currents, while reducing the repolarizing potassium ones, leading to a prolongation of the refractory period [49]. Moreover, cells like fibroblasts and myofibroblastsresiding in EAT are capable ofcoupling with myocardiocytes via gap junction formation, leading to the depolarization of their resting membrane potential [50]. These changes, along with the abovementioned fibrosis, increase the anisotropy of atrial tissue and impair atrial conduction. The duration of the P wave and PR segment, as well as the P wave dispersion, have all been found to positively correlate with the amount of EAT [51,52,53]. Furthermore, the studies that used electroanatomical mapping reported that EAT volume was positively correlated with the area of complex fractionated atrial electrograms [54]. Given these changes, it is not surprising that people with AF present significantly higher levels of EAT compared to those with normal sinus rhythm [55]. 

The strategy of early rhythm control in patients with newly diagnosed AF was associated with fewer adverse cardiovascular events when compared to the rate control strategy in EAST-AFNET 4 trial [56]. The choice between interventional treatment and anti-arrhythmic drugs for maintaining sinus rhythm was welladdressed in CABANA trial, where catheter ablation reduced both arrhythmia recurrence and the composite of death or cardiovascular hospitalization [57] Nevertheless, it is estimated that about 30% of patients undergoing pulmonary venous isolation will experience AF recurrence [58]. Thus, it is essential to better identifythose who will benefit more from ablation and to investigate the reasons behind treatment failure. 

Even though our findings are not sufficient to modify the current clinical practice, they provide several interesting implications for future research. Being the largest analysis, it strengthens our knowledge regarding the role EAT in post-ablation AF recurrence, proposing a potential role of peri-LA EAT in the pathophysiology of AF recurrence. Thus, it could serve as a target for the so-called upstream therapies, which use non-anti-arrhythmic agents to reverse atrial remodeling and further reduce recurrence [59]. In a population of patients undergoing coronary artery bypass grafting, an injection of botulinum (a toxin that suppresses gaglionated plexi activity) in EAT reduced post-operation AF [60]. Additionally, regular exercise and common anti-diabetic medications, as well as statins, have all been found to decrease the volume of EAT [61,62,63,64,65,66]. Some of these interventions, like metformin [67] and regular exercise [68], have been associated with a reduced risk ofrecurrence after catheter ablation. Whether these favorable effects can be attributedat least in part to the reduction inEAT remains to be investigated by future well-designed proof-of-concept studies. Similarly, whether therapies that could specifically target peri-LA EAT reduction may influence AF ablation outcome is also subjected to future research. 

On the other hand, we found that total EAT volume did not differ significantly between patients with and without recurrence. This observation may be of special interest, as it may reflect some pathophysiological insights. Inour opinion, this finding highlights the importance of the local role of EAT. One hypothesis may be that at least in patients with already established AF, its maintenance might be associated mainly with the regional alterations which peri-atrial EAT confers. Local paracrine activity, along with the remodeling induced by the direct fibrofatty infiltration and the electrical connection between EAT and myocardiac cells, as described above, may play a key role in the explanation of our findings. This hypothesis is supported by the findings of Gaborit et al. who investigated the genome of EAT samples collected from different places. They reported significant differences between samples collected from the atriae when compared to those collected from EAT surrounding the ventricles and the coronary arteries, concluding that there is a specific transcriptomic signature of EAT which depends on its location [69].

## 5. Limitations

This study has also some limitations. First, most of the studies were of retrospective nature and were judged as beingmoderate quality. Moreover, we were not able to perform diagnostic accuracy meta-analyses due to data unavailability. Additionally, older, overweight, non-Asian patients, as well as those with persistent AF and those who underwent cryoballoon ablation, were under-represented in this analysis; consequently, no robust conclusions couldbe drawn for these subgroups. Furthermore, no details regarding the distribution of EAT around the LA were available for additional analyses, while both analyses seemed to suffer from heterogeneity, which could not be adequately explained by the subgroup and meta-regression analyses. Finally, the included studies lacked information about the pathophysiological background of AF. It is known that many comorbidities such as hyperehyperthyroidism [70], as well as lifestyle factors such as smoking and physical activity [71,72], are potentially reversible causes of AF development. On the other hand, conditions like channelopathies [73] are not treatable. Nevertheless, the current analysis cannot explore the confounding effect of different pathophysiological backgrounds on the relationship between epicardial fat and AF recurrence. Thus, it is important to highlight that common comorbidities associated with AF recurrence [74,75,76,77,78,79,80,81] should be properly treated, irrespectively of epicardial fat. 

## 6. Conclusions

Only peri-LA EAT seems to be associated with AF recurrence following catheter ablation, with higher volumes decreasing the chance to maintain normal sinus rhythm. Interestingly, total EAT does not seem to be associated with the ablation outcome. Limited literature data, further corroborated by our findings, support that behind these observed differences may lie the location-specific genome of the EAT, but this remains a matter of future research. Moreover, given that cardiac computed tomography is routinely performedprior to ablation for the characterization of pulmonary venous anatomy, it would be of clinical interest whether peri-LA EAT could play a prognostic role. More studies are needed for the derivation of a cut-off value which might identifypatients who will benefit more from transcatheter pulmonary venous isolation. Finally, as there is an ongoing interest in therapies that target EAT reduction, it should be further investigated whether this pathophysiological mechanism could help our effort to reduce post-ablation AF recurrence. 

## Figures and Tables

**Table 1:** Summary table of included studies.

Study	N	Males (%)	Age (y)	BMI	FU (m)	PAF (%)	HT (%)	DM (%)	CT METHOD	PVI METHOD	Comments
Nagashima et al. 2011 [25]	40	77.5	58 (10.2)	23 (2.6)	10.2	60.0	37.5	n/a	SA. HU: −50 to −200	RF^+^	Endpoint: AF BP: 2 m AADs: < 6 m
Tsao H. M. et al. 2011 [26]	64	81.3	54.6 (8.5)	25.6 (3.3)	7.6	60.9	15.6	10.9	SA. HU: −50 to −200	RF^+^	Endpoint: AF/AT BP: 3 m AADs < 8 w
Nakahara S. et al. 2014 [27]	60	83.3	61.1 (10.4)	n/a	16	35.0	78.3	n/a	SA. HU: −50 to −200	RF^+^	Endpoint: AF/AT BP: 3 m AADs: <6 m
Nakatani Y. et al. 2015 [28]	55	74.5	64 (9)	24 (2.7)	12	47.3	52.7	12.7	SA. HU: −50 to −200	RF^+^	Endpoint: AF BP: 3 m AADs: < 3 m
Masuda M et al. 2015 [29]	53	67.9	61 (11)	24.2 (3.2)	16	41.5	49.1	18.9	SA. HU: −50 to −200	RF^+^	Endpoint: AF/AT BP: 3 m
Singhal R. et al. 2016 [30]	32	84.4	58 (10)	25 (3)	17	71.9	56.3	12.5	SA. HU: −50 to −200	RF^+^	Endpoint: AF BP: 3 m AADs < 8 w
Monno K. et al. 2018 [31]	104	72.1	63 (10)	23.9 (3.7)	22	55.8	52.9	20.2	SA. HU: −50 to −200	RF^+^/CRYO	Endpoint: AF BP: 3 m
Kawasaki M. et al. 2019 [20]	64	50	70.6 (9)	23.7 (3.7)	11	100	56.3	3.1	SA. HU: −45 to −195	RF^-^/CRYO	Endpoint: AF/AT BP: 3 m AADs: < 3 m
Nakatani Y. et al. 2020 [32]	44	84.1	64 (10)	25 (3)	21	59.1	52.3	6.8	SA. HU: −50 to −200	RF^+^	Endpoint: AF BP: 3 m AADs: no restriction
Hammache N. et al. 2021 [33]	389	65.8	58.1 (11.1)	27 (4.7)	12	100	40.1	7.2	SA. HU: −50 to −250	RF^-^	Endpoint: AF BP: 3 m AADs: 3 m
Beyer C. et al. 2021 [19]	732	73.2	57.6 (10.8)	26.9 (4)	31	88.4	46.2	4.1	SA. HU: −45 to −195	RF^-^/CRYO	Endpoint: AF BP: 3 m
Goldenberg G. R. et al. 2021 [34]	130	68.5	61 (5)	28.7 (4)	19.5	67.7	60.8	26.9	SA. HU: −45 to −195	CRYO	Endpoint: AF/AT BP: 3 m
Yang M. et al. 2022 [35]	251	59.0	62 (9)	25 (3)	12	68.9	54.2	13.9	SA. HU: −50 to −200	CRYO	Endpoint: AF/AT BP: 3 m AADs: < 8 w
Jian B. et al. 2022 [36]	337	53.4	55.2 (12.1)	25.3 (2.2)	12	n/a	n/a	15.7	automate. HU: −50 to −200	RF^-^	Endpoint: AF/AT BP: 3 m
Yang M. et al. 2022 [37]	680	63.8	62.5 (9)	24.59 (3.1)	12	0	50.9	14.7	SA. HU: −30 to −190	CRYO	Endpoint: AF/AT BP: 3 m AADs: < 8 w

AADs: anti-arrhythmic drugs. AF: atrial fibrillation. AT: atrial tachycardia. BMI: Body Mass Index. BP: blanking period. CRYO: cryoablation. DM: diabetes mellitus. FU: follow up. HT: hypertension. HU: Hounsfield units. m: months. n/a: not available. PAF: paroxysmal Atrial Fibrillation. PVI: pulmonary venous isolation. RF+: radiofrequency ablation with additional lesions beyond PVI. RF^-^: only PVI radiofrequency ablation. SA: semi-automated. w: weeks. y: years. Continuous variables are summarized as mean (SD).

## Data Availability

Data will be available upon reasonable request.

## Supplementary Materials

The following supporting information can be downloaded at: https://www.mdpi.com/article/10.3390/jcm12196369/s1, Figure S1: Funnel plot for identification of publication bias in studies evaluating total epicardial adipose tissue; Figure S2: Funnel plot for identification of publication bias in studies evaluating peri- left atrium epicardial adipose tissue; Figure S3: Forest plot of pre- ablation total epicardial adipose tissue standardized mean difference between patients with and without AF recurrence, by grouping studies according to the method used for pulmonary venous isolation; Figure S4: Forest plot of pre- ablation peri- left atrium epicardial adipose tissue standardized mean difference between patients with and without AF recurrence, by grouping studies according to the duration of follow up; Figure S5: Forest plot of pre- ablation peri- left atrium epicardial adipose tissue standardized mean difference, between patients with and without AF recurrence. Only studies that assessed both total and peri left atrium epicardial adipose tissue have been included. Figure S6: Forest plot of pre- ablation total epicardial adipose tissue standardized mean difference, between patients with and without AF recurrence. Only studies that assessed both total and peri left atrium epicardial adipose tissue have been included; Table S1: Quality assessment tool used for evaluation of the included studies; Table S2: Quality score of the included studies.
