# Supplementary material for: Epicardial Adipose Tissue and Atrial Fibrillation Recurrence following Catheter Ablation: A Systematic Review and Meta-Analysis
Source: J Clin Med. 2023 Oct 5;12(19):6369. doi: 10.3390/jcm12196369 (PMC10573952; doi:10.3390/jcm12196369)

SUPPLEMENTARY MATERIAL

TITLE: Epicardial Adipose Tissue and Atrial Fibrillation Recurrence following Catheter Ablation: A Systematic Review and Meta-Analysis

AUTHORS: Ioannis Anagnostopoulos [1,*], Maria Kousta [1], Charalampos Kossyvakis [1], Nikolaos Taxiarchis Paraskevaidis [1], Dimitrios Vrachatis [2], Spyridon Deftereos [2] and Georgios Giannopoulos [3]

[1] Cardiology Department, Athens General Hospital "G. Gennimatas", 11527 Athens, Greece
[2] 2ndDepartment of Cardiology, National and Kapodistrian University of Athens, 15772Athens, Greece
[3] 3rd Department of Cardiology, Aristotle University of Thessaloniki, 54124Thessaloniki, Greece
* Correspondence: iannis.anagnostopoulos@gmail.com; Tel: +30-2107768132

Supplementary Table S1 Quality assessment tool used for evaluation of the included studies

CRITERIA	YES (2 points)	CD/NA/NR (1 point)	NO (0 points)
**1. Objective**			
1.1 Was the research question/objective in the study clearly stated exploring the association between epicardial fat and recurrence of atrial fibrillation after catheter ablation?			
**2. Sample**			
2.1Were eligibility/selection criteria for the study population prespecified and clearly described?			
2.2Was the participation rate of eligible persons at least 50%?			
2.3Were all the subjects selected or recruited from the same or similar population, including the same time period? Were inclusion and exclusion criteria for being in the study prespecified and applied uniformly to all participants?			
2.4 Was the sample size justification, power description or variance and effect estimated provided?			
**3. Outcome reporting**			
3.1 Were the epicardial fat measured prior to the recurrence of atrial fibrillation being measured?			
3.2 Were the timeframe sufficient so that on could reasonably expect to see an association between epicardial fat and recurrence of atrial fibrillation after catheter ablation if it existed?			
3.3Did the study examine different ranges of epicardial fat as related to the risk of atrial fibrillation?			
3.4Were the epicardial fat measures clearly defined, valid, reliable and implemented consistently across all study participants?			
3.5 Was the amount of epicardial fat assessed more than once over time?			
3.6Was the incidence of atrial fibrillation clearly defined, valid, reliable and implemented consistently across all study participants?			

3.7 Were the atrial fibrillation assessors blinded to the epicardial fat status of participants?			
3.8Was loss to follow-up after baseline 20% or less?			
3.9 Were key potential confounding variables measured and adjusted statistically for their impact on the relationship between epicardial fat and risk of atrial fibrillation recurrence (OR/HR)?			

CD: cannot determine, NA: not applicable, NR: not reported

Supplementary Table S2 Quality score of the included studies

Study	1.1	2.1	2.2	2.3	2.4	3.1	3.2	3.3	3.4	3.5	3.6	3.7	3.8	3.9	Overall %
Tsao H. M. et al 2011	2	1	1	1	0	2	1	2	2	2	2	1	2	0	67.9
Nagashima K. et al 2011	2	1	1	2	0	2	1	2	2	2	2	1	2	0	71.4
Nakahara S. et al 2014	2	0	1	1	2	2	1	2	2	0	2	1	2	2	71.4
Nakatani Y. et al 2015	1	2	1	1	0	2	1	0	2	2	2	1	2	0	60.7
Masuda M. et al 2015	1	2	1	2	0	2	2	2	2	0	2	1	2	2	75
Singhaul R. et al 2016	1	1	1	1	0	1	1	2	2	2	2	1	2	2	67.9
Monno K. et al 2018	1	1	1	1	0	1	1	2	2	2	1	1	2	2	64.3
Kawasaki M. et al 2019	2	2	2	2	2	2	1	2	2	1	1	2	2	2	89.3
Nakatani Y. et al 2020	2	2	1	2	0	2	2	2	2	2	2	2	2	2	89.3
Goldenberg G. et al 2021	2	2	1	1	0	2	2	2	2	2	2	2	2	2	85.7
Beyer C. et al 2021	2	2	1	1	0	2	1	2	2	2	1	2	2	0	71.4
Hammache N. et al 2021	2	2	2	2	0	2	1	2	2	0	2	2	2	2	82.1
Yang M. et al 2022	2	2	2	2	0	2	1	2	2	2	2	2	2	2	89.3
Jian B. et al 2022	2	2	2	2	2	2	1	0	2	0	2	1	2	2	78.6

**Yang M. et al 2022**	2	2	2	2	0	1	1	0	2	2	2	2	2	2	78.6

Supplementary Figure S1 Funnel plot for identification of publication bias in studies evaluating total epicardial adipose tissue

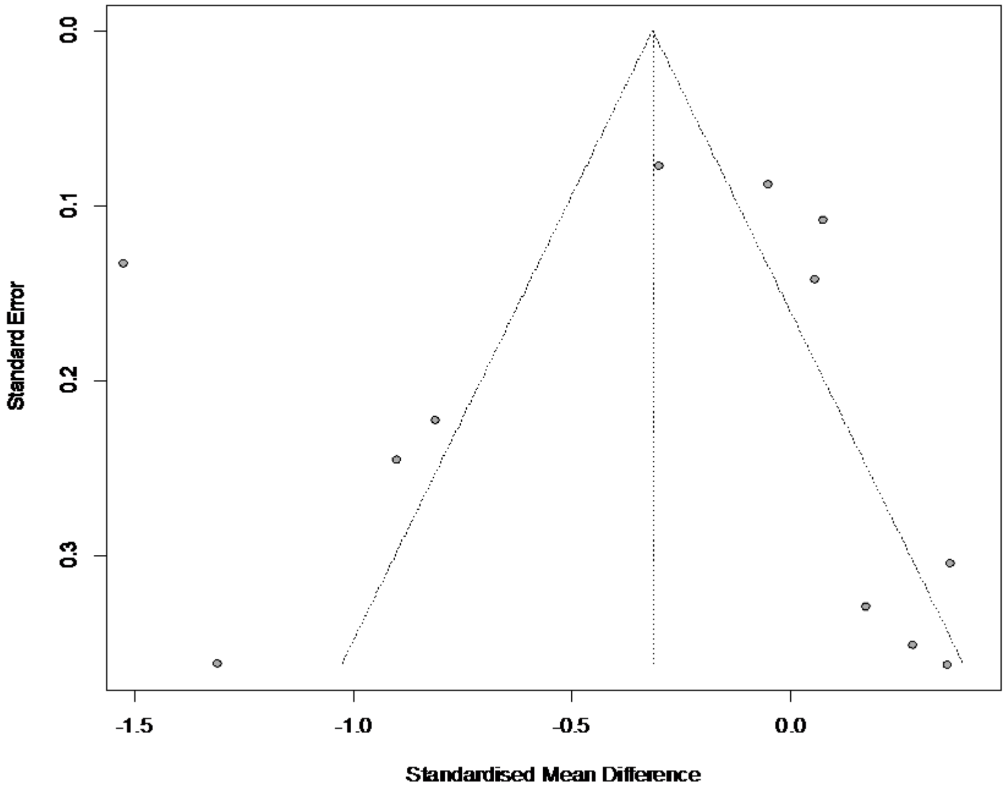

Supplementary Figure S2 Funnel plot for identification of publication bias in studies evaluating peri- left atrium epicardial adipose tissue

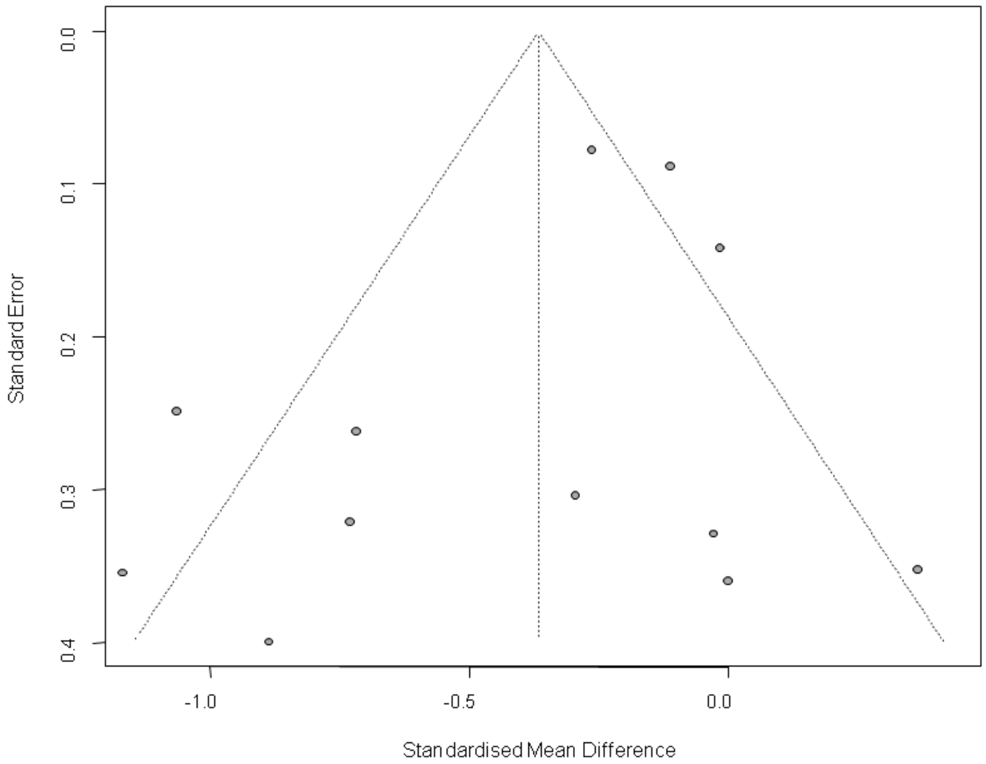

Supplementary Figure S3 Forest plot of pre- ablation total epicardial adipose tissue standardized mean difference between patients with and without AF recurrence, by grouping studies according to the method used for pulmonary venous isolation.

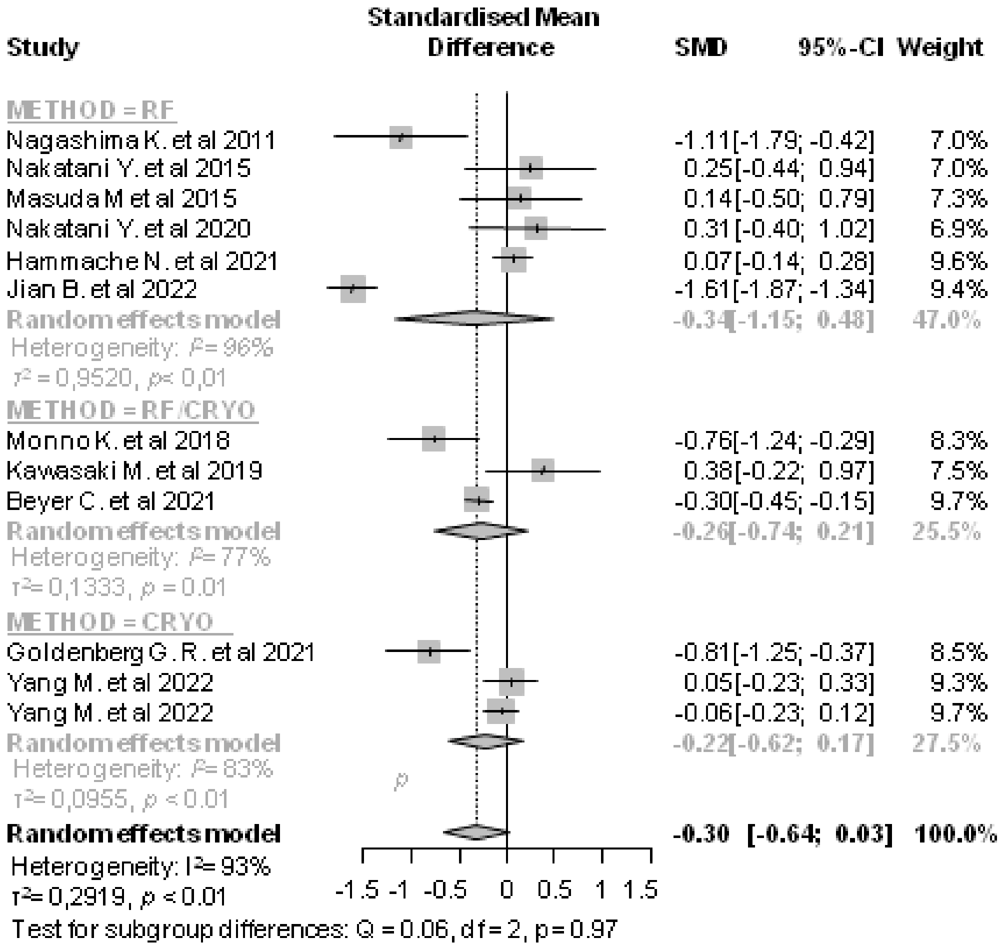

Supplementary Figure S4 Forest plot of pre- ablation peri- left atrium epicardial adipose tissue standardized mean difference between patients with and without AF recurrence, by grouping studies according to the duration of follow up.

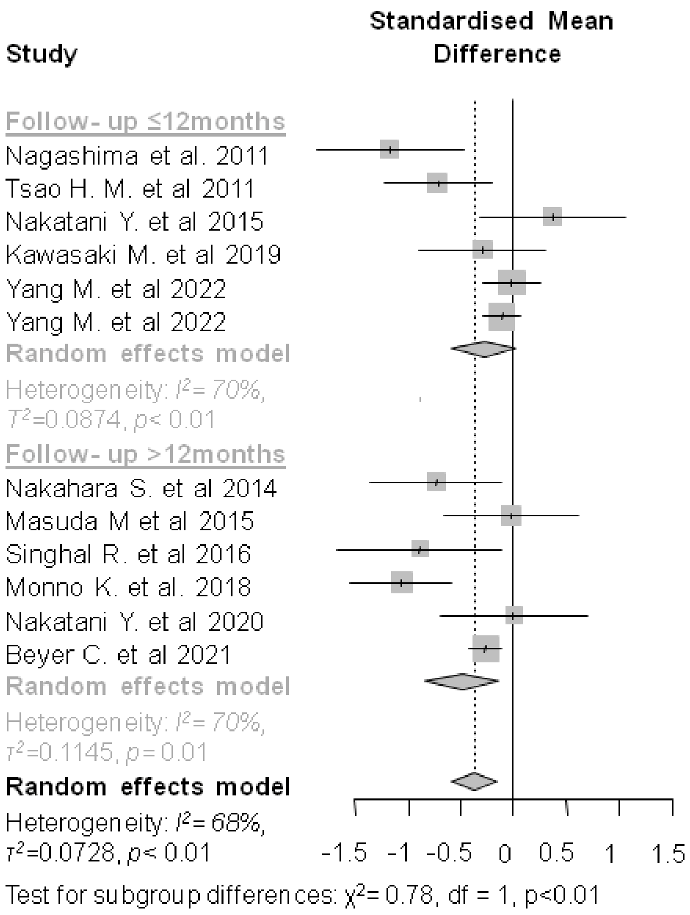

Supplementary Figure S5 Forest plot of pre- ablation peri- left atrium epicardial adipose tissue standardized mean difference, between patients with and without AF recurrence. Only studies that assessed both total and peri left atrium epicardial

adipose tissue have been included.

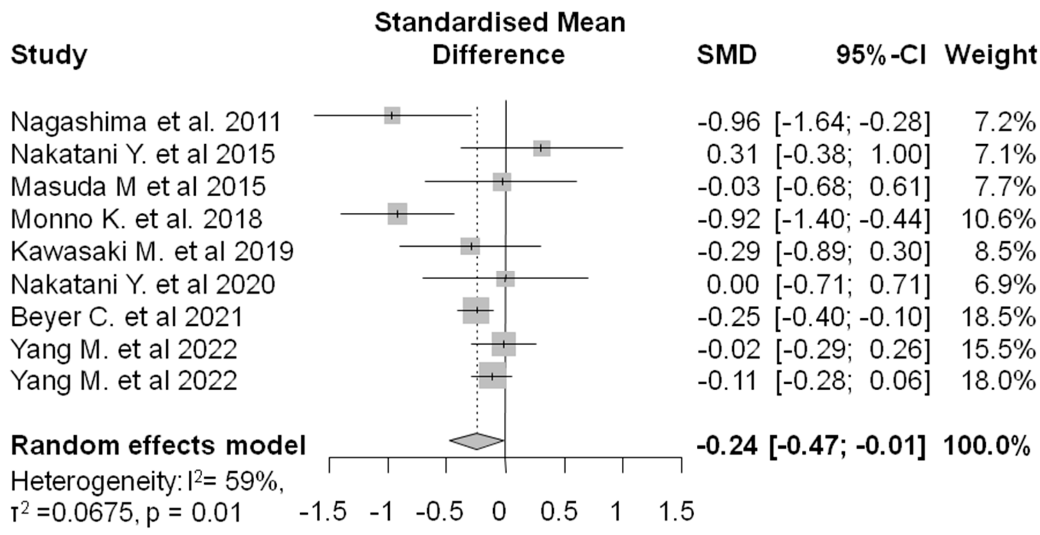

Supplementary Figure S6 Forest plot of pre- ablation total epicardial adipose tissue standardized mean difference, between patients with and without AF recurrence. Only studies that assessed both total and peri left atrium epicardial adipose tissue have been included.

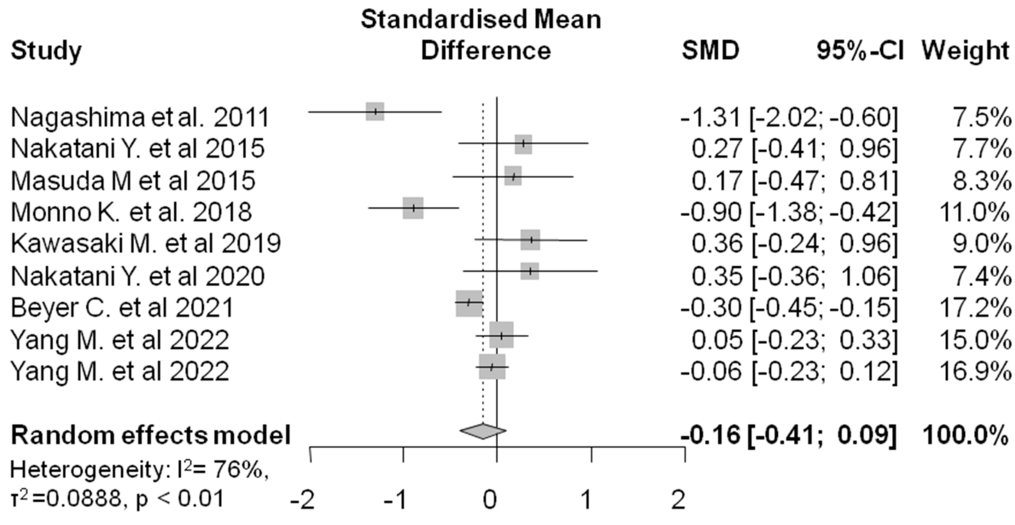

Supplement: Supplementary file 1 [file jcm-12-06369-s001.zip › jcm-2628998-supplementary.pdf]
